# “Removing an Ogiek from the Forest is like removing a fish from water”: A qualitative examination on Ogiek community impacts from forced land eviction for conservation

**DOI:** 10.1371/journal.pgph.0004460

**Published:** 2025-06-25

**Authors:** Mau Forest, Daniel M. Kobei, Samson L. Luari, Danya Carroll, Samrawit Gougsa, Victoria Pratt, Nicole Redvers

**Affiliations:** 1 Ogiek Peoples’ Development Program, Egerton, Kenya; 2 Schulich School of Medicine and Dentistry, University of Western Ontario, London, Canada; 3 Minority Rights Group, London, United Kingdom; 4 The Hub at Wellcome Collection, London, United Kingdom; 5 Invisible Flock, West Bretton, United Kingdom; 6 Arctic Indigenous Wellness Foundation, Yellowknife, Canada; PLOS: Public Library of Science, UNITED STATES OF AMERICA

## Abstract

Despite evidence that Indigenous Peoples are better guardians of their Forests than international or state protection agencies, they continue to be forcibly evicted from their Lands. Additionally, despite the known impacts that forced land eviction for conservation has on the well-being of Indigenous Peoples in varied contexts, there remains limited appreciation of the well-being impacts of forced land eviction within Kenya from an Indigenous perspective. With this, the aim of this research was to better understand the well-being impacts of forced land evictions from an Ogiek perspective. Twenty-six semi-structured interviews and one sharing circle (*n* = 7) were carried out with Ogiek Peoples in Kenya between December 2021 and March 2023. The interviews and sharing circle were transcribed verbatim, then reflexive thematic analysis was carried out through iterative coding to identify key themes. Six themes were characterized including: 1) Our cultural practices, ceremonies, and spirituality are tied to our identity as Ogiek; 2) Our foods and plants are our medicines; 3) Maintaining our culture in an everchanging world; 4) The Forest and Ogiek are as one in a reciprocal relationship; 5) Removing an Ogiek from the Forest is like removing a fish from water; and 6) Hope that our rights will be recognized. Findings also demonstrated that the forced displacement of Ogiek Peoples by government entities has continued to impact the social and economic vitality of their communities. Our study exemplifies the substantial and ongoing impacts of colonial conservation approaches on Ogiek Peoples in Kenya, and highlights the continued need for local and international allies to stand in solidarity with and support Ogiek and other Indigenous Peoples in their efforts to return as the original stewards of their Forest and other homelands.

## Introduction

Indigenous Peoples globally have stewarded their traditional territories for millennia. It is well documented that Indigenous Peoples can effectively steward varied and valuable ecosystems which benefit all people, communities, and the planet [1–3]. Forests stewarded by Indigenous Peoples, for example, have been demonstrated to hold more carbon reserves than forests stewarded by non-Indigenous Peoples [1]. Furthermore, Indigenous Peoples’ territories represent much of the remaining ecologically intact landscapes globally [2], and their knowledges are necessary for combatting biodiversity loss [3]. Despite Indigenous Peoples’ historical and ongoing leadership roles in effective land stewardship, as well as factors such as climate change, colonial conservation efforts (including non-Indigenous led carbon credit schemes), and the ongoing infringement on Indigenous Peoples’ Land rights, their role in stewardship continues to be threatened [4]. Additionally, there is continued marginalization and devaluing of Indigenous Peoples and their worldviews [5]. This devaluing includes the Indigenous Traditional Ecological Knowledges (ITEK) Indigenous Peoples hold that ensures the continued relational-based stewardship of Mother Earth [6]. This marginalization and devaluing of Indigenous Peoples and their knowledges in varied regions of the world additionally leads to ongoing human rights violations (e.g., violence, forced land eviction) and land abuses (e.g., unconsented resource extraction). One example of this marginalization and devaluing of Indigenous Peoples is the experiences of the Ogiek Peoples as described below.

Many Indigenous Peoples in Africa, including Ogiek Peoples in Kenya, have close relationships and ties with their Forests. Ogiek Peoples are East Africa’s largest community of hunters and gatherers [7] and have lived within the Mau Forest Complex (MFC) for millennia. Ogiek ways of being and ways of life has long fostered their economic, sociopolitical, and cultural structures which are “solely dependent on the [F]orest” [7]. Ogiek Peoples are also very effective hunter-gatherers, important stewards of Bees, and holders of deep and expansive Traditional Ecological Knowledges related to their Forest landscapes. Ogiek knowledge has fostered cultural and traditional environmental management systems that have been pivotal to the long-standing stewardship of the Mau Forest. Forest management has always been a part of Ogiek responsibility to protect Lands, Rivers, and traditional foods. Traditionally, Ogiek clans were each assigned different Land areas that they were responsible for protecting [7]. Despite these important Forest protection roles, Ogiek Peoples have continued to be subjected to human rights violations and unjust land grabs, including forced land evictions, that have been happening from British colonization [7] all the way to the present day [8].

There continues to be a disconnect in some Euro-Western-centric conservation communities on what constitutes equitable and “sustainable” conservation, and at what cost it should come to those living within areas thought to be in need of “conservation” [9]. Ogiek Peoples are and have been at the forefront of the impacts of colonial conservation mindsets and approaches, as well as the strategies used by government to displace those who have lived on their lands for millennia. With this, despite broad local evidence that Indigenous Peoples are “better guardians of their forests than international or state protection agencies,” they continue to be forcibly evicted from their Lands [10]. As noted, Ogiek Peoples continue to face forced and often violent land evictions, with land displacement originally happening during initial British colonization and then again occurring from the 1970s onwards at the hands of the Kenyan government. The Ogiek Peoples’ Development Program (OPDP) is an Ogiek-led non-governmental organization that was established in 1999 by Ogiek Elders, leaders, farmers, and professionals due to high levels of marginalization, including land dispossession [10]. OPDP honors Ogiek Peoples’ culture, inclusion, and works to promote and protect Land rights, including environmental protection and overall sustainable development [10]. In November 2009, OPDP, the Centre for Minority Rights Development (CEMIRIDE) and Minority Right Group International (MRG) filed a case that was later referred to the African Commission on Human and Peoples Rights (ACHPR) regarding Ogiek land grievances [11,12]. In the 45^th^ session of the ACHPR, they delivered a landmark ruling in favor of the Ogiek community in 2017 [11]. Although the OPDP effort in the ACHPR was successful, the ruling has been disregarded by the Kenyan government. With this, there continues to be inaction by the Kenyan government and eviction of Ogiek communities continues to occur as recently as November 2023, with community members left out in the rain with no immediate governmental supports for resettlement. The most recent forced eviction of the Ogiek community in 2023 has been thought to be connected to carbon markets, with the Kenyan government seizing the opportunity for taking “control over an increasingly lucrative asset” [8].

The ongoing forced land evictions have had monumental and detrimental consequences on Ogiek culture and livelihoods. The land evictions have additionally created an intergenerational rift in Ogiek knowledges, with land displacement causing challenges in the passing down of Traditional Knowledge relevant for the protection and sustainment of the Mau Forest Complex for future generations. Although forced land displacement of Indigenous Peoples has been documented to have substantial impacts on the health and well-being of Indigenous Peoples in varied contexts globally [13], including in Africa [14], there remains limited appreciation of the well-being impacts of forced Land eviction within Kenya from an Indigenous perspective. With this, to date there has never been ‘Ogiek-led research’ carried out to investigate the local views of the individual and collective impacts of forced land eviction on Ogiek communities. Therefore, the overarching aim of the current research was to better understand Ogiek Peoples’ perspectives on the well-being impacts of forced land evictions for conservation.

### Positionality

Any research related to Indigenous Peoples and/or their knowledges are increasingly expected to have positionalities declared amongst the author team [15–17]. “Nothing about Indigenous Peoples, without Indigenous Peoples” [18], is an important tenant relevant to all Indigenous-related work. With this, we position ourselves here within this work.

**Fig 1:**
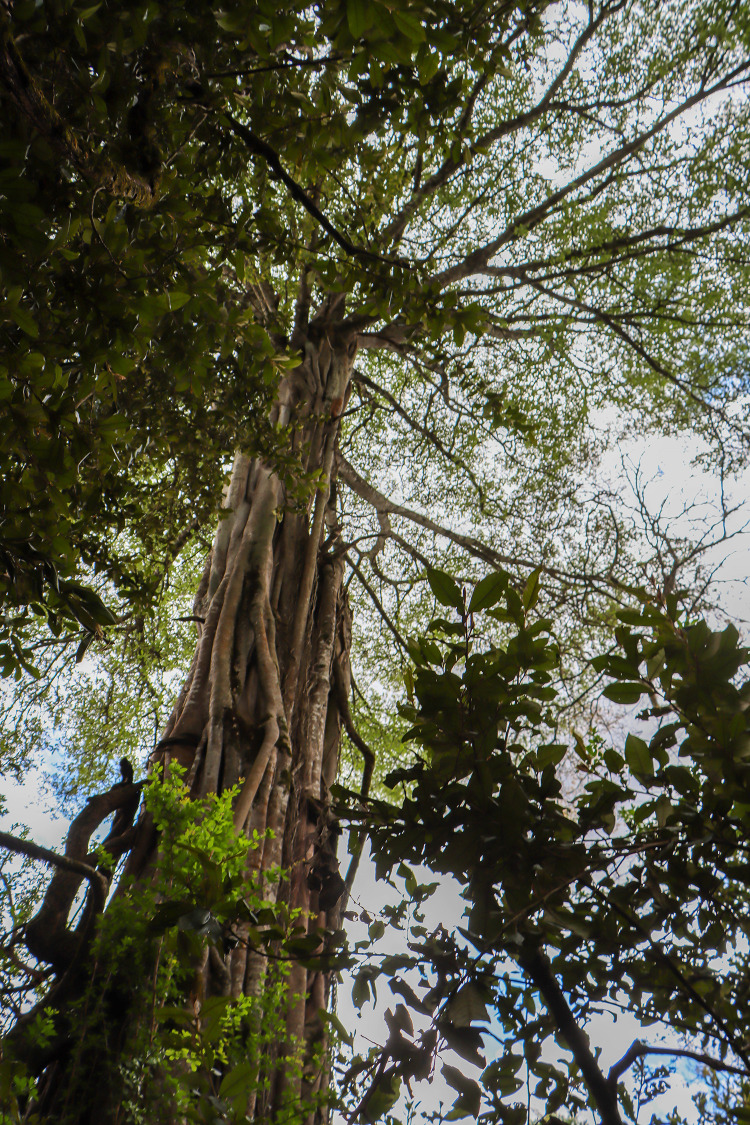
The Mau Forest stewarded by Ogiek Peoples from time immemorial. *Republished from an unpublished collection under a CC BY license, with permission from the Ogiek Peoples Development Program (OPDP), original copyright 2024.

DMK is a member of the Ogiek community, as well as the founder and current Director of the Ogiek Peoples Development Program (OPDP). DMK is a longtime Indigenous Peoples’ activist operating at regional, national, and international levels. SL is a youth member of the Ogiek community, and a member of the OPDP team. DC is a Diné and White Mountain Apache invited Indigenous health scholar from the United States providing capacity building and research-related technical support. SG and VP are non-Indigenous allies based in the UK providing capacity-building assistance through the Land Body Ecologies network (LBE). NR is a member of the Deninu K’ue First Nation in the sub-Arctic region of Canada, and is an invited Indigenous and planetary health scholar providing research technical support. We would also like to formally acknowledge our first author, the Mau Forest (see Fig 1), who is seen as an entity in her own right with a voice platformed here through the Rights of Nature. In addition to the deep cultural and spiritual significance to Ogiek Peoples, the Mau Forest Complex has a massive ecological significance, including water storage, flood mitigation, protection of biodiversity, carbon-sequestration, and regulation of micro-climates in the region [7]. Water originating from the Mau Forest Complex supports more than four million people in Kenya and northern Tanzania [7]. Given this, we formally honor the Mau Forest in this work. Additionally, we have capitalized words such as Forest and Land purposely throughout this article to recognize the significance of these Mother Earth–based relatives, as is common in Indigenous writing styles [18].

## Materials and methods

### Research questions

This research was entirely led by an Ogiek community-based research team. At the beginning of the project, the research team came together with outside collaborators and allies to develop research priorities and research questions relevant to the Ogiek community. Appropriate research questions were therefore co-developed by the Ogiek community-based research team with external collaborators invited by the community. With this, the research questions for this work were: 1) What does the Forest mean to us as Ogiek Peoples, and how do we depend on the Forest? and 2) What have been the impacts on our Ogiek community due to the ongoing forced evictions from our Forest homelands?

### Overall study design

A qualitative research study was developed and led by Ogiek community members (DMK, SL), with collaborators providing Indigenous research-related technical support (DC, NR), as well as capacity building from LBE (SG, VP). The overall research was embedded within a decolonial theoretical framework that ensured a “collaborative process of naturalizing Indigenous intent, interactions, and processes and making them evident to transform spaces, places, and heart [19].” To better ensure an optimal environment based on the local context for sharing within the Ogiek language, semi-structured interviews were determined to be the most appropriate method of data collection as well as a sharing circle [19] with youth (as explicitly requested by Ogiek youth). The interviews and sharing circle also allowed for the flexibility of sharing story narratives as well as using a “deep listening” approach [20] within the data collection process, which is relevant for Indigenous research methodological approaches. With this, the decolonial approach to sharing (whether through interviews or the sharing circle) was seen to be “one” from an Indigenous holistic approach, which meant the data collected was combined. Although some researchers may combine different qualitative methods for practical or pragmatic considerations [21] (despite inherent limitations well described in the literature [22]), our Indigenous holistic approach to viewing the data collected was consistent with an Indigenous form of triangulation, where the members of the community are seen to be so closely connected as relations that it would be inappropriate in this context to separate them. Despite this, we also carried out additional reflection in the results section on places where there might have been variance to the views shared.

The research received ethics approval to carry out this work from the St. George’s Research Ethics Committee (#2021.0230). Additionally, approval was gained for this research in January 2022 from the Ogiek Council of Elders, registered as “Gotop Sogoot.” Under the United Nations Declaration on the Rights of Indigenous Peoples (UNDRIP) [23], Indigenous Peoples have collective rights to self-determination including the freedom to pursue their own economic, social, and cultural development. With this, as leaders of their community, the Ogiek Council of Elders holds the authority to provide local approval of the work independent of any university ethics body. Standards for reporting qualitative research (SRQR) were followed for this work [24].

### Setting

Ogiek Peoples consist of over 45,000 individuals who are traditionally located in East Kenya, primarily in the Mau Forest Complex, which is the largest Indigenous Forest block in Kenya spanning across 400,000 hectares of land [25]. The Mau Forest Complex is about 200 km from the capital Nairobi. The research described in this article was carried out in the Nakuru and Narok Counties of Kenya. More specific geographic information is not provided to ensure the anonymity of research participants given the small population of Ogiek Peoples.

### Recruitment and consent

The research team initially introduced the project to the OPDP board members before informing the rest of the Ogiek community through forums and workshops, including those being held at the Ogiek Cultural Center. Ogiek of Mau were specifically recruited for participation in this study, with Ogiek of Mau communities mapped out ahead of time to facilitate an easier recruitment process. The recruitment focus on the Ogiek of Mau was due to this community wanting to develop, lead, and carry out such a project. After informing the community, and gaging interest, the OPDP leadership called community leaders in each area to engage them on who would be able to speak to the topic (as is common practice in Indigenous-led research). The OPDP team would then call prospective participants by phone, and if they did not have a phone, they would call their relatives to inform them of the project. Project information was passed on verbally due to this being the most common method of communication, and which also avoided literacy barriers. The community and potential participants were given a period of six months after the initial project introduction before they were reengaged in the research project to express interest and give consent for participation.

Many Ogiek community members in the Mau areas impacted by land eviction were known to the OPDP leader, which enabled a level of trust with prospective participants. Most of the community was aware of past and current work done by the OPDP team, which enhanced understanding of the relevance of the research project and its potential for articulating their concerns around land issues. Since the project aimed to gather information from Ogiek community members who have been impacted by forced land evictions, purposive sampling [26] was engaged through established local networks of the Ogiek research team. Snowballing also occurred with participants recommending other potential participants in their communities. OPDP staff followed up with recommended individuals to assess their interest in participating in the study. Participants were able to therefore participate if they were members of the Mau Ogiek community, were over the age of 18, and were from areas that had been forcibly evicted. There was particular attention given to ensure recruitment of women and Elders given their key roles as caregivers and knowledge keepers in the community. Youth community members (18–35yo) were also included given their unique experiences and challenges with land eviction affecting intergenerational knowledge transfer.

Once participants agreed to participate in the research, they were consented verbally by the Ogiek research team. They were informed that they could cease participation at any time in the research. Consent forms were translated into both the Ogiek and Swahili languages. The consent form was reviewed in the language preferred by the participant, and the consent recorded. No incentives were provided to participants for participation in the research, which was decided to be the best approach by community leaders in the region. If participants were unable to travel, the research team would travel to their location. No participants withdrew from the research study or declined participation in the study.

### Interviews and focus group data collection

Semi-structured interviews were carried out by Ogiek research team members which included one male and one female. The interviews were carried out in the Swahili (*n *= 3) and Ogiek (*n* = 23) languages (with language preference determined by the participant) between December 2021 and March 2023. The interviews lasted between 30–70 minutes and were audio recorded. Those carrying out the interviews with participants did not know the participants prior to the interview. One of the interviews involved interviewing two participants at the same time as was requested by the participants. The semi-structured interview guide was developed by the OPDP staff and included open-ended questions on topics including, as examples, views on the relationship between the participant and the Forest; views on how they see their culture continuing into the future; perspectives on the forced land eviction and its impact on their well-being. In addition to the interviews, a sharing circle was held with youth participants aged 18–35 years old (*n* = 7), who expressed their preference of being able to share their perspectives in a group setting. Group settings can be more culturally responsive to social norms and allowed for youth participants to participate on their own terms [27].

During the interviews some participants became emotional given the difficult topic. Participants were provided cultural supports if desired, and were offered to take breaks or cease participation if desired. Many participants shared with the interviewers afterwards that they felt a sense of relief after sharing despite the difficulty in talking about some aspects of the eviction, as they never had a chance to tell their story before this.

### Data analysis

Interviews were transcribed verbatim by OPDP staff from the Swahili and Ogiek languages into English. The transcripts were then further anonymized to ensure no community-identifying information was included given the small population size. Some Indigenous language words were not able to be translated into English as there was no known English word (e.g., name of a specific traditional food from the Forest). Another OPDP leader and advanced language holder was then brought in to re-review all of the audio recordings and the transcripts to ensure they were accurately translated. Interview transcripts were then uploaded to NVivo (Release 14) qualitative software for analysis. Reflexive thematic analysis outlined by Braun and Clark [28], and advanced by Liebenberg and colleagues [29] was carried out to identify key themes. Collaborative data analysis was carried out in this project from coding to the characterization of themes tracked through folders on NVivo for audit purposes. The collaborative analysis involved the direct involvement of a local Ogiek youth in the data analysis for capacity building purposes as well as to better ensure an Ogiek lens was applied to viewing the coding and theme characterization within the appropriate cultural context (SL). This approach allowed space for honoring the Ogiek perspective and the ways in which the research was approached and interpreted [29]. Two authors (SL, DC) each carried out preliminary inductive coding of all the transcripts, while a third author (NR) was brought in for discussion, as well as for the refinement of the codes and themes. Debriefs were held throughout the coding process to ensure a reflexive practice was enabled including with a fourth author (DMK). Supporting participant quotes were identified to demonstrate the themes that were ultimately characterized.

## Results

Twenty-six semi-structured interviews and one sharing circle *(n* = 7) were conducted between December 2021 and March 2023. There was a diversity of males and females represented among the participants, and ages that were volunteered ranged from 24 to 83 years of age. Due to cultural considerations, if a participant did not want to provide their age this was respected; however, given this, there is a possibility that there may be ages outside of the above noted age range. Other demographic information is not shared on participants due to the small region and risk of identification.

Through reflexive thematic analysis, six themes were characterized from the data (see Table 1). Overall, the characterized themes encompassed the importance of cultural identity and Land connections among Ogiek Peoples as well as the impacts of forced land eviction on Ogiek lifeways, culture, knowledge, and health and well-being. Overviews of the characterized themes are described in detail further below.

**Table 1:** Main themes characterized from the data.

Themes
Our cultural practices, ceremonies, and spirituality are tied to our identity as Ogiek
Our foods and plants are our medicines
Maintaining our culture in an everchanging world
The Forest and Ogiek are as one in a reciprocal relationship
Removing an Ogiek from the Forest is like removing a fish from water
Hope that our rights will be recognized

### Our cultural practices, ceremonies, and spirituality are tied to our identity as Ogiek

Important elements of Ogiek culture were discussed by participants, including their ways of knowing, their connection with the Forests, and their spirituality’s role in their Peoples’ past and future. Several participants expressed the importance of spirituality in daily life. There were also, however, discussions on the changes to belief systems, including the introduction of Christianity to the community.

*The Ogiek knew the existence of a God. The Elders prayed early in the morning facing the rising sun. I think it is the same God we pray to as Christians but the way we traditionally prayed was different (ID 4007)*.

The intergenerational transfer of knowledge was stated to be significant for Ogiek Peoples. Storytelling was noted to facilitate this knowledge transfer of valuable knowledge, including the principles of coexistence and respect for the Forest that is taught to younger generations. Participants shared that stewardship of the Forest is taught through Ogiek stories including the balance that must be maintained among their Tree relatives.

*Children were taught stories and dances, but for now things are changing slowly. Stories related to the Forest because it was important to the Ogiek. Cutting was done carefully and not every tree was cut. These stories were passed from the Elders to young people (ID 4011).*

Participants articulated through the interviews that there is an inextricable link between Ogiek and the Forest. They provided examples of how the Forest has provided sustenance for Ogiek Peoples since time immemorial. Furthermore, they shared how the Forest has also provided a sacred space where Ogiek Peoples are able to teach their children through Land-based education systems that have existed for millennia. One participant expressed this sentiment on the role of the Land in raising their children.

*…[W]e used the Land to farm and feed our children. We depended on the Land to educate our children. We still practice our tradition and culture (ID 4012).*

Participants expressed that in recent decades there has been a transition away from traditional lifestyles among Ogiek Peoples to more modern systems that have created barriers to accessing and transferring cultural knowledge. This transition coupled with forced land evictions from their traditional territories have impacted their ability to practice their culture and preserve their Traditional Knowledge that is connected to the Forest.

*Our culture has been lost since our avenues to practice our culture was not accessible anymore. We no longer have places to assemble and practice our culture. Places such as [place] is no more because we have no place to build (ID 4016).*

Furthermore, the disconnection with Land and culture (from being evicted from the Forest) began in the younger years of some participants when they had just started learning their Land-based cultural education. This generation and following generations were stated to not have the time in the Forest that their ancestors and parents had. Some participants expressed that there is a

*…lack of cultural knowledge among the group and other youths that can be attributed to the fact that after they were evicted, their parents and grandparents no longer had time to teach them about culture, instead they were focused on getting daily bread (ID 4031).*

A feeling of disconnection with the culture and Land was expressed by many of the participants, including Elders and youth. This disconnection was stated to have many consequences on Ogiek lifeways, ceremonies, cultural knowledge, health, and well-being. The time when government evictions began for Ogiek Peoples was also stated to mark a time when the loss of a society and culture built on stewardship for the Forest began. When Ogiek Peoples were forcibly removed from the Forest, it was noted that they were no longer able to care for the Forest as their Peoples have for millennia. To some participants the eviction meant that the Forest itself also had become more vulnerable and that,

*…our hearts feel sad when we see the Forest is being destroyed (ID 4017).*

### Our foods and plants from the Forest are our medicines

For Ogiek, the Forest represents a space where they can access plants and herbs that have provided health, medicine, and healing for their Peoples for millennia. Participants shared that plants from the Forest produce foods such as honey (with help from the Bee relatives) that not only represent nutritional value but also provide cultural and economic value such as for trading for other needed commodities. Ogiek are stated to be the original Bee keepers as they followed Bees in the Forest and stored honey for times when food was scarce.

*…During the drought, the stored honey was eaten to sustain the community to the next season when honey is ripe (ID 4005).*

Ogiek were additionally noted to have an immense respect for the life-sustaining role that Trees provide for their Peoples. Trees and plants from the Forest were stated to provide medicinal properties that Ogiek Peoples used when needed.

*It is painful to see people destroying trees and shrubs that are our medicine. They do not understand the value of most of the species within this Forest. They recklessly destroy everything without a care. The damage they cause is without measure (ID 4033).*

It was described by some participants that traditionally, herbal medicine was often the only means of treating ailments and diseases among Ogiek Peoples, and these medicines came from the Trees in the Forest.

*We don’t want anyone to destroy any Tree because it is our rescue for herbal medicine for expectant women, our children, and other herbs to maintain our health as Ogiek because we don’t have access to hospitals. Our hospital is herbal medicine (ID 4021).*

It was stated that Ogiek Peoples understand that the value of the Forest, Trees, and other non-human relatives goes well beyond their monetary value. Participants shared how the value of the Forest is embedded in a deep indescribable interconnected relationship that Ogiek Peoples have had with the Forest for millennia. A majority of the participants described the cultural and spiritual value of the Forest to their Peoples and their concern for the damages that have been done to the Forest since the forced land evictions began. Participants additionally all shared a respect and responsibility for the Forest as well as concern for how the Forest itself has also been impacted by the forced land evictions. They expressed how the damage to the Forest that has been caused by outside entities have and will continue to have harmful impacts on Ogiek Peoples and their Forest relatives (e.g., animals and plants).

### Maintaining our culture in an everchanging world

There were mixed perceptions among participants about whether lifestyle changes in their communities have been mostly negative. Some participants expressed that the education their children have been able to access now has been beneficial.

*It is just recent that education was introduced to our children and we accepted the change. We thank God for this change and we hope it will help boost our children (ID 4009).*

Other notable changes that were highlighted by participants as a result of government interference in Ogiek affairs included more restrictions on accessing traditionally hunted foods such as wild game. Several participants stated that they must now abide by laws that do not allow hunting.

*Our lifestyle as a community has changed and this has changed our relationship with the Land and Forest. I used to depend on the Forest alone as a Bee keeper and hunter. Now I no longer depend on these activities as hunting is illegal and Bee keeping is no longer enough to feed my family (ID 4007).*

Participants also shared that they were concerned that there continues to be a loss of culture and knowledge in their community. They expressed that they were once a nomadic Indigenous Peoples but due to having their children attend school as well as the Forest and land access issues they cannot move as freely as they once did.

*As Ogiek, we did move to zones within our territories in the Mau Forest when honey is ripe in certain areas for example in [name]. We followed Bees for honey and nowadays we don’t move because our children are in school hence limiting our movement. Hence, we don’t want the Forest destroyed (ID 4017).*

Many of the participants shared the need to preserve their cultural knowledge and connection with the Forest despite changes that have been forcibly imposed on their communities. They expressed the need to maintain their culture in an ever-changing world is tantamount to preserving their identity and connection with the Forest. This connection with their Forest was stated to be the cornerstone of how Ogiek Peoples have effectively stewarded the Forest areas in Kenya for generations.

### The Forest and Ogiek are as one in a reciprocal relationship

Ogiek Peoples’ relationship with the Forest was stated to be a strong example of Indigenous stewardship. Participants shared that this relationship based on reciprocity and respect has sustained Ogiek Peoples and their Forest relatives for millennia. Ogiek have always been a part of the Forest and the Forest a part of them as expressed by many participants.

*The Forest and Ogiek are one and we talked directly with the Forest (ID 4008).*

Living within the Forest for millennia, Ogiek Peoples were stated to have mastered the balance needed to ensure that their communities had all that they needed without exceeding their limits. Participants shared that the Forest has always provided for their communities; however, due to the forced land evictions and other changes, this delicate balance has been undermined.

*The Ogiek community used to live totally dependent on the Forest resources. As every member of the community had access to these resources, there was neither a poor nor a rich person within the community (ID 4007).*

Ogiek conservation as expressed by participants represents a decolonial approach to preserving and stewarding the Forest. Despite this long-established record of being the original Forest conservationists, participants asserted that Ogiek Peoples have not been recognized by local governments at the levels needed to ensure their rights to continue their role as key conservationists.

*The Ogiek were great conservationists, it is just farming that brought destruction (ID 4015).*

Participants also expressed concern that the intentions of governments and conservationist groups have not been beneficial for the Forest. The colonial approach to conservation was stated to be more harmful to the Forest than beneficial. Furthermore, the disconnect between other entities and the Forest, including the lack of a relationship with the Forest, has been observed by some participants.

*People who are employed to protect Forests are the ones aiding in its destruction as they do not care like we do about these Forests. They have just been dispatched here from other parts of the country. They have no connection to these Forests. For them protecting Forests is just work, for us it is a way of life. I wish the government could consider us and allow us to guard our heritage. They could use us instead of bringing outsiders to our Lands (ID 4033).*

Participants shared that they still do want to continue their role as stewards and guardians of the Forest; however, they have been and continue to be overlooked by local governments.

*We are in our Forest, the Ogiek Forest. Us as Ogiek do not entertain destruction of the Forest (ID 4021).*

### Removing an Ogiek from the Forest is like removing a fish from water

An exhaustive narrative of injustices was stated to have been imposed upon Ogiek Peoples, including multiple forced land evictions from their home territories. Some participants stated that they have lived through many of these forced land evictions, and that they have been devastating and destabilizing to their families and communities. One participant stated that the land evictions have caused immense stress and grief.

*This is the second eviction after we were evicted in 2005. We lost everything and our livelihood. I am stressed a lot. When I see my Land I cry a lot (ID 4012).*

Another participant shared that their family was forcibly and violently removed from their home and Land. There was noted to be a realization and a lack of hope that stemmed from the fact that they were not only removed from their home, but also now were forced to seek help from those that had evicted them (i.e., the government). The experiences shared by participants on the forced removal of themselves and their families and communities underscore the many human rights violations that were stated to have been experienced by Ogiek Peoples. It was noted that many, if not all, of those who were forcibly evicted were given a moment’s notice by the government to leave their homes and traditional territories. These notices were stated to have had and continue to have traumatic impacts on those directly affected.

*When the notice elapsed, they found us still living in the Forest; we had not moved. They removed us by force. They found me at home and beat me mercilessly. I watched as they burnt my house…My children were scared when they heard guns going off, some ran into the Forest, we had to look for them later. Our sheep also got scared and ran into the Forest too. We lost hope because we realized that the government we were going to seek help from is the same government that had evicted us (ID 4007).*

Participants also shared that there was a complete disregard by government entities that had evicted them from their Lands on where their communities would be placed after the forced land evictions. The government entities were stated to not have a plan on where to put their communities, nor did they consider the vast consequences that would result from the forced land evictions—including on mental health.

*When we were evicted we were never told where to go and the fencing commenced and they never minded our welfare while we had school-going children and what to feed our children because we had nowhere to till… People became stressed, we lost our people through depression due to this effect (ID 4008).*

The forced land evictions have had numerous additional impacts on Ogiek Peoples as shared by participants. Participants expressed that there has been a lack of recognition of the rights of Ogiek Peoples to live their lives in the Forest. Removal of their Peoples from their Forest territories was noted to be devastating as they have always lived in the Forest. A few of the participants shared that they can relate with a fish that has been removed from water.

*I feel so bad because we were never given justice. We were tortured and never recognized as Ogiek. Removing an Ogiek from the Forest or from his territory is like removing a fish from water (ID 4008).*

Evictions were stated to be continuing to occur among Ogiek communities. Participants expressed that these evictions mark a time in Ogiek history when their Peoples have been disconnected from their traditional territories. Participants shared that the generations that have lived through the forced evictions have been removed from the lands of their ancestors and hollowed grounds where they have buried their Peoples.

*We are the group that were evicted in 2019 from our Land. We were evicted and forced to live on the roads with no Land to live. Our great-grandparents died in this Land. We are Ogiek and this was our territory. This government forcefully disregarded our rights (ID 4009).*

Participants conveyed how ongoing evictions of Ogiek Peoples have marginalized their Peoples in all areas of life, health, and well-being. Ogiek Peoples were stated to be once strong in their culture, stewardship, and traditional lifeways and now have been forced into places where they cannot learn or apply their knowledges. Some participants linked this marginalization with being subjected to poverty and disadvantage.

*As Ogiek, we have been marginalized and disadvantaged in all spheres of life and development. We were evicted and we are landless now (ID 4017).*

Many of the participants expressed the feeling of not having a home or land base due to the forced land evictions. As shared by participants, this overall reality of being landless has had serious cultural and well-being implications and consequences for Ogiek communities.

### Hope that our rights will be recognized

Despite the numerous injustices that have been experienced by Ogiek Peoples, there was still stated hope that they will be restored to their original lands. Many participants shared the importance of having hope that through legal processes and advocacy their rights will be recognized by the Kenyan and local governments.

*For now, we have some hope that our rights will be recognized by the government (ID 4011).*

Participants also expressed that while they are concerned for their Peoples they are also concerned for the Forest and their non-human relatives. Participants were fully aware of the damages and destruction that have also occurred in the Forest due to the forced land evictions. One participant stated that there cannot be anymore destruction of the Forest if its ecosystems are to remain intact.

*As Ogiek group in [name], we don’t want any more destruction. We want the Forest to remain intact so that it can feed us (ID 4017).*

Finally, there were some participants who called out the government on its negligence in protecting the Forest. One participant voiced a strong statement calling out various levels of government, including international entities, to stand in solidarity with Ogiek Peoples and their fight to live and protect their Forest home.

*The county government is not protecting our Forest. We call the government to protect our Forest because the authorities in charge have not really protected as expected. We call the international community and the president of Kenya…so that he can deliver our ruling in Arusha and leave corruption which has delayed our case for a long time, 10 or even 20 years (ID 4022).*

It was stated that much needed work remains to support Ogiek Peoples in their continued fight for being able to return to their original traditional territories in the Forest.

### Reflections on the youth voice

Although youth were seen to be interconnected to the deep listening process engaged in this research, we provide space here to elevate some of the voices shared by this group. Youth participants shared similar views on protecting and conserving their Forest homelands. The intergenerational connection of stewardship was particularly apparent in the sharing by the youth participants. One youth participant shared that they felt it was vital that the Forest will be conserved and available for them and their generation to protect and learn from.

*I speak as a youth I would say that we want the Forest conserved and protected (ID 4022).*

Despite living through numerous changes, youth participants shared that they wanted to actively contribute to conserving and protecting their Forest areas. One youth participant shared:

*As [the] Ogiek community love and have the knowledge to protect Forests, we should be engaged… to help in protecting it…Youths could be trained and given the mandate to protect the Forest (ID 4033).*

Youth participants voiced their similar concerns of witnessing outsiders destroy the Forest. Several participants noted how the destruction of the Forest has affected them.

*Right now, we feel pain as we watch them [name] neglect protecting the Forests. We see them allowing people to bring power saws into the Forests. These machines are very noisy and wild animals run further into the Forests to avoid this noise (ID 4033).*

Ogiek youth participants also expressed their concern for changes that they have observed in the environment, including less rainfall and hotter and longer drought seasons. One participant stated:

*Now we are witnessing forest fires constantly. Various forces are destroying our Forest and I am afraid with time there will be no Forest to talk about (ID 4032).*

Similar to the older adult participants, youth participants also shared their deep pain of being evicted from their homes and Lands. One participant stated that,

*…[w]e were hopeless as we lost everything within the span of a day. We woke up rich and slept poorly. Everything was destroyed. People took advantage to loot everything from us (ID 4033).*

## Discussion

*We want recognition…as [the] Ogiek community. We want our home, and [to be]given the right to live within our territory because we don’t have an alternative land to call home (ID 4025).*

The interviews and sharing circle in this study provided important insights into the impacts of forced land evictions and displacement among Ogiek communities. Six overarching themes were characterized including: 1) Our cultural practices, ceremonies, and spirituality are tied to our identity as Ogiek; 2) Our foods and plants are our medicines; 3) Maintaining our culture in an everchanging world; 4) The Forest and Ogiek are as one in a reciprocal relationship; 5) Removing an Ogiek from the Forest is like removing a fish from water; and 6) Hope that our rights will be recognized. The participants in this study provided many examples on how the forced land evictions have impacted their community structures (kinship structures), cultural continuity, and overall way of life. Our findings also demonstrated that the forced displacement of Ogiek Peoples by government entities has continued to impact the social and economic vitality of their communities. In the Forest, Ogiek Peoples were able to provide for their families and communities through hunting, trading, and other avenues. The forced land evictions, however, have restricted Ogiek access to their Forest and therefore their ability to live cooperatively, maintain their traditional livelihoods, and continue to steward the Forest for all. Ogiek Peoples have been forced to transition from being fully self-sufficient to having to depend on outside entities for any type of welfare and support. To date there has been no support provided by the Kenyan government for displaced Ogiek Peoples. The forced reliance on outside entities (including government) is not unlike what has happened in other regions where Indigenous Peoples were forcibly removed from their traditional homelands. For example, in the United States, “federal policies promoted settler-colonialism land theft, and forced removal of Native nations, which disrupted…food systems and lifeways, contributing to present-day food insecurity and poor health outcomes” [30].

As demonstrated by the most recent land eviction of Ogiek Peoples in November 2023 [8], Indigenous Peoples continue to be forcibly removed from their Land by government entities around the world [31–33]. The human-rights based impacts of forced land evictions have been increasingly appreciated; however, the health and well-being as well as the cultural impacts have been less appreciated. To our knowledge, aside from this current study highlighting the voices of Ogiek Peoples in Kenya, there has only been one other ‘Indigenous-led’ study examining the impacts of forced land evictions on Indigenous Peoples in the African context. With this, Batwa Peoples in Uganda have experienced very similar human rights violations and atrocities, having also been forcibly removed from their traditional territories by the Ugandan government for conservation purposes [34]. A recent Batwa-led research study had some similarities with the Ogiek study presented here in that there was noted interconnectivity with their Forest for physical, mental, emotional, and spiritual well-being that was detrimentally impacted by land eviction [14]. There was also similar sentiment on the risk and community consequences of being disconnected from their traditional territories for intergenerational knowledge transfer and the need for Land rights to be upheld to ensure Indigenous stewardship of the Forest [14].

Colonial and “fortress” conservation continues to therefore threaten Indigenous Peoples’ livelihoods and customary Land rights worldwide, including for Ogiek and Batwa Peoples [13]. Land evictions such as those imposed upon Ogiek Peoples are a fundamental threat to their survival as they, like many other Indigenous Peoples, depend on access to their ancestral lands for cultural survival [13]. Although both Ogiek and Batwa Peoples were forcibly removed under a conservation agenda (not unlike other Indigenous communities) [34], Indigenous Peoples are forcibly removed and dispossessed from their lands for other reasons around the world, including for tourism (inclusive of big game hunting) [35], green energy infrastructure, and for industrial resource development (e.g., logging and farming) which has impacted Indigenous Peoples in North and South American contexts as well as in Nordic countries (Norway & Finland), Australia [36], and many other regions [32]. Land dispossession and displacement therefore continues to impact Indigenous Peoples globally despite their proven roles as stewards and guardians of their traditional territories [1–3]. Given this, the Ogiek Peoples experiences outlined in this research are unfortunately one of many examples of human rights violations within the international context. What is unique to this research, however, is that it was led by Ogiek Peoples themselves.

Our findings also highlighted the intimate relationship that Ogiek Peoples have with their Forest areas and non-human relatives (such as plants and bees). The relational worldview that underpins this relationship is often missing in colonial conservation approaches in Africa and other contexts, where conservation can be argued to be an extension of the legacy of colonization. Prior research has demonstrated that there are numerous benefits to non-colonial conservation practices, including through Indigenous Land guardian programs such as those in Canada [4] and Australia [36]. For example, in Canada, Indigenous guardian programs are funded by the federal government to provide funding to Indigenous Peoples to exercise responsibility in stewardship of their traditional lands, waters, and ice [37,38]. The guardian initiative “supports Indigenous rights and responsibilities in protecting and conserving ecosystems, developing and maintaining sustainable economies, and continuing the profound connections between natural landscapes and Indigenous cultures” [38].

Certain regions of the world, including in Africa, have been particularly slow in recognizing the conservation value of keeping Indigenous Peoples on their traditional homelands. Indigenous Peoples, such as Ogiek Peoples, have the ability to contribute to protecting and safeguarding the Forest areas as they have since time immemorial. Indigenous-led Land guardian programs can provide mechanisms for intergenerational knowledge transfer, improve the economic and social stability of Indigenous communities, ensure the maintenance of the Forest, and most importantly keep Indigenous Peoples connected to their traditional territories. Indigenous Peoples know their landscapes the best. As one participant in this study stated: *“How can an outside[r] protect something that they do not know its value nor care for its prosperity*? *We have been pushed aside to allow [name] officers to protect our heritage, but instead we have witnessed its destruction. I feel pain as there is nothing I can do about it. There is a feeling of helplessness among us now (ID 4026).”* An Indigenous-led Land guardian program funded by local governments for Ogiek Peoples to continue to be the caretakers of the flora and fauna of their Forest while still maintaining full access for livelihood and cultural use can have numerous benefits for both humans, the Mau Forest, as well as her non-human inhabitants.

It is additionally important to highlight from our findings how the Mau Forest herself has been impacted by the forced eviction of Ogiek Peoples. Many participants articulated that they are concerned about how the forced land evictions have impacted the Forest, including how the Forest has now become more vulnerable to being destroyed by extractive industries. Common conservation framing has often included a militarized approach that antagonizes and criminalizes Indigenous Peoples as Peoples needing to be removed from their traditional territories [13]. This approach has also been used by the Kenyan government as reasoning for the evictions of Ogiek Peoples, including portraying the narrative that Ogiek were damaging the Forest and therefore must be removed. Our findings clearly demonstrate the reverence, care, and respect for the Forest that Ogiek Peoples have, as well as the recognized importance that the Mau Forest must remain intact and be protected for future generations of Ogiek. These research findings are therefore counter to current government narratives.

What is often not considered, however, as noted, is due to the relational nature of Indigenous Peoples and their landscapes, it is also the Forest that suffers as well when its Indigenous caretakers are removed from it. The importance of Indigenous stewardship to maintain Forest health and well-being has been well documented in other regions. For example, prior research has shown that Indigenous Peoples in some regions of the world have benefited the health of Forests through traditional land-management practices such as cultural burning [39]. Colonial policies that were put in place limited and criminalized traditional fire management resulting in a deterioration of the Forest ecosystems that were dependent on cultural fire regimes [39]. More recent recognition of Indigenous Forest management practices in some regions of the world have led to increasing use, including with cultural burning, with value recognized for enhancing climate change adaptation and preparedness for communities in and near Forests [40]. Governments and other actors in Africa, Asia, and other regions have been very behind in recognizing the importance of Indigenous Peoples for the health of Forests and other land bases.

Overall, this study highlights the continued need for local and international allies to stand in solidarity with and support of Ogiek and other Indigenous Peoples in their efforts to return as the original stewards of their Forest homelands. Land evictions and dispossession continues to impact Ogiek Peoples and many other Indigenous Peoples worldwide. Non-Indigenous entities, including national and international conservation organizations, must recognize that these types of land-grab evictions significantly impact the health and well-being of Indigenous Peoples, as well as limits the ability for intergenerational Traditional Ecological Knowledge (TEK) transmission to occur, which ultimately impacts planetary health for all [41]. Conservation organizations could consider making funding available to country offices contingent on a recognition and respect for Indigenous Land rights, and with funding pulled for the forced land evictions of Indigenous Peoples.

Land evictions, including those that continue to impact Ogiek Peoples, are an infringement on their human rights, including those outlined in UNDRIP [23], with, in the Ogiek case, a clear violation of Article 10 of UNDRIP with no free, prior, and informed consent to relocate. Despite this encroachment on Ogiek Peoples ability to preserve their cultural knowledge and way of life, they remain vigilant. The OPDP recently celebrated the opening of the Ogiek Cultural Center in Nkareta, Narok County, Kenya in March 2024. This is one step for cultural preservation; however, it will only go so far without having the Land rights and the access to the Forest to ensure applied knowledges are passed on to future generations.

### Limitations

Despite a strong effort to ensure broad representation of Mau Ogiek communities, our study may not fully be representative of all Ogiek Peoples and communities who have experienced forced land eviction. Despite this, we are confident that the data presented from the region gives a good idea of some of the main important points relevant to the topic given how cross-cutting the findings were across ages, genders, and communities. Our data collection process was done in the Ogiek and Swahili languages. Indigenous languages such as the Ogiek language, are verb-based and have very different language structures than the English language which is noun-based. Given this, some words are just not translatable in the same way. With this, some of our participant quotes being translated into the English language may be missing some of the nuances relevant to the Ogiek language with regards to the depth of meaning. Despite this, we have made substantial efforts to ensure the meaning embodied in the sharing by participants has been reflected as accurately as possible. This process has been better enabled by having co-authors that speak both Ogiek and English fluently. Lastly, qualitative research in general can be limited in terms of the transferability of the findings to other contexts. Given the dearth of available research regarding Indigenous Peoples and Africa in the context of their well-being as a result of forced land eviction, it is difficult to appropriately assess the potential for transferability of our study. As noted in the discussion section, however, there appears to be some similarities with other contexts, which makes us think there are insights worth considering for other regions.

## Conclusion

*…we call that the Ogiek community to be given mandate to live and conserve their Forest (ID 4024).*

This research exemplifies the substantial impacts of colonial conservation approaches on Ogiek Peoples in Kenya. The voices and perspectives of those directly impacted by land evictions including Ogiek Peoples are underappreciated and they continue to be marginalized by national and local governments. This Ogiek-led research project contributes an Indigenous perspective that is lacking in local and national contexts in Africa. Participants in this study provided valuable insights on their experiences and the impacts from forced land eviction on the overall individual and collective well-being of their communities. Ogiek worldviews and knowledges around Forest conservation and stewardship are significant for maintaining the health of the Mau Forest areas in Kenya—especially amid the climate crisis. There is a continued need for local, national, and international governmental recognition of Ogiek rights to their traditional territories and Forests. Returning to and having access to their traditional territories is necessary not only for the current existence of Ogiek Peoples but also for future generations of Ogiek Peoples and the world. Indigenous Lands in Indigenous hands (i.e., LandBack [42]) has the “potential to ameliorate national and global shortfalls in land protection for biodiversity conservation…” [43] and improve overall planetary health.

## Supporting information

S1 Checklist (PDF)
